# Effects of Gestational and Postnatal Exposure to Chronic Intermittent Hypoxia on Diaphragm Muscle Contractile Function in the Rat

**DOI:** 10.3389/fphys.2016.00276

**Published:** 2016-07-12

**Authors:** Fiona B. McDonald, Eugene M. Dempsey, Ken D. O'Halloran

**Affiliations:** ^1^Department of Physiology, School of Medicine and Medical Science, University College DublinDublin, Ireland; ^2^Department of Paediatrics and Child Health, Cork University Maternity Hospital and the Irish Centre for Fetal and Neonatal Translational Research, University College CorkCork, Ireland; ^3^Department of Physiology, School of Medicine, University College CorkCork, Ireland

**Keywords:** development, early life stress, perinatal, respiratory muscle, hypoxia

## Abstract

Alterations to the supply of oxygen during early life presents a profound stressor to physiological systems with aberrant remodeling that is often long-lasting. Chronic intermittent hypoxia (CIH) is a feature of apnea of prematurity, chronic lung disease, and sleep apnea. CIH affects respiratory control but there is a dearth of information concerning the effects of CIH on respiratory muscles, including the diaphragm—the major pump muscle of breathing. We investigated the effects of exposure to gestational CIH (gCIH) and postnatal CIH (pCIH) on diaphragm muscle function in male and female rats. CIH consisted of exposure in environmental chambers to 90 s of hypoxia reaching 5% O_2_ at nadir, once every 5 min, 8 h a day. Exposure to gCIH started within 24 h of identification of a copulation plug and continued until day 20 of gestation; animals were studied on postnatal day 22 or 42. For pCIH, pups were born in normoxia and within 24 h of delivery were exposed with dams to CIH for 3 weeks; animals were studied on postnatal day 22 or 42. Sham groups were exposed to normoxia in parallel. Following gas exposures, diaphragm muscle contractile, and endurance properties were examined *ex vivo*. Neither gCIH nor pCIH exposure had effects on diaphragm muscle force-generating capacity or endurance in either sex. Similarly, early life exposure to CIH did not affect muscle tolerance of severe hypoxic stress determined *ex vivo*. The findings contrast with our recent observation of upper airway dilator muscle weakness following exposure to pCIH. Thus, the present study suggests a relative resilience to hypoxic stress in diaphragm muscle. Co-ordinated activity of thoracic pump and upper airway dilator muscles is required for optimal control of upper airway caliber. A mismatch in the force-generating capacity of the complementary muscle groups could have adverse consequences for the control of airway patency and respiratory homeostasis.

## Introduction

Alterations to the supply of oxygen during early life pose a profound stress to physiological processes during critical periods of development, with potentially deleterious outcomes that can be long-lasting. Sleep-related breathing disorders are characterized by intermittent pauses in breathing during sleep with associated blood oxygen desaturations. Obstructive sleep apnea (OSA) is recognized as a common condition in adults, but more recently there is increased focus on the incidence of OSA in childhood and during pregnancy that has long been overlooked. Apnea of prematurity is a recognized phenomenon characteristic of preterm babies. Chronic intermittent hypoxia (CIH) is a central feature of disordered breathing and is increasingly recognized as a major driving force in the development of a wide range of morbidities, including maladaptive remodeling in cardiorespiratory control (O'Halloran, 2016). The early life environment should prepare offspring for long-term success and it is therefore important that developmental conditions do not produce a mismatch with the adult environment. Physiological adaptations to perturbations during development may sustain life before birth or in early life, but may come at a cost in terms of biological trade-offs, with deleterious consequences for homeostatic regulation in later life.

The physiological changes that occur during pregnancy predispose pregnant women to the development of OSA (Loube et al., 1996; Izci et al., 2006). Gestational OSA has been identified in many recent studies as a major clinical problem. In the absence of large scale longitudinal studies, the prevalence of gestational OSA remains unclear, but evidence of gestational OSA has been reported in several studies (Edwards et al., 2005; Chen et al., 2012; Pien et al., 2014) and may be more common in women with higher body mass index (Ko et al., 2012; Louis et al., 2012; Sarberg et al., 2016). Gestational OSA is likely to result in pre-placental oxygen desaturations whereby both the mother and her fetus become hypoxic. Maternal O_2_ desaturations have been shown to coincide with decelerations in fetal heart rate (Sahin et al., 2008), despite the greater oxygen-carrying capacity of fetal hemoglobin, which left-shifts the hemoglobin-oxygen dissociation curve, which means oxygen loading occurs at lower oxygen concentrations in the fetus. Recent studies report that mothers with sleep-disordered breathing had increased evidence of uteroplacental hypoxia and fetal distress (Bourjeily et al., 2015a,b; Ravishankar et al., 2015). Studies to date show that gestational CIH may be responsible for some of the poor fetal outcomes following gestational OSA. Gestational CIH is reported to cause reductions in fetal growth (reversible), increased normoxic ventilation, attenuated peak hypoxic ventilatory response (short-term), and accentuated hypoxic ventilatory depression in postnatal pups, as well as glucose dysregulation (Gozal et al., 2003; Iqbal and Ciriello, 2013; Iqbal et al., 2014).

Preterm birth is associated with aberrant control of breathing owing to amongst other things, immaturity in the central respiratory networks governing respiratory control. Periodic breathing with recurrent central apneas are common and are associated with repeated exposure to (IH Razi et al., 2002; Martin et al., 2011; Di Fiore et al., 2013; Decima et al., 2015). Several studies have reported long-lasting deleterious effects of exposure to hypoxia during critical periods of the postnatal period in rodents (Okubo and Mortola, 1988; Gozal et al., 2002; Reeves et al., 2003, 2006). Notwithstanding the critical role of respiratory muscles as the final effector in the respiratory control system there is a general paucity of information concerning the short- and long-term effects of exposure to hypoxia during development on respiratory muscle function and respiratory mechanics. There are no studies to date exploring the effects of early life exposure to CIH on diaphragm function. This represents an important gap in our knowledge, of potential clinical relevance. The aim of this study was to investigate the effects of exposure to gestational CIH and postnatal CIH on diaphragm muscle function in male and female rats. Both male and female animals were studied given the evidence of male susceptibility to early life stressors (Zeitlin et al., 2002; Mage and Donner, 2006; Johnston and Hagberg, 2007), notwithstanding our previous observation that there are no sex differences in the effects of pCIH on sternohyoid muscle force (McDonald et al., 2015b, 2016).

## Methods

### Ethical approval

All experiments described herein were performed under license from the Irish Government, Department of Health and Children in accordance with National and European legislation following approval by the University College Dublin animal research ethics committee.

### Mating

Male and female Wistar rats were housed together overnight in wire bottomed cages; trays beneath the cage were checked the following morning for evidence of a copulation plug, which was taken as a sign of successful mating.

### Exposure to chronic intermittent hypoxia (CIH)

Animals were exposed to either gestational CIH (gCIH) or postnatal CIH (pCIH) using commercial chambers in which the environmental oxygen concentration was carefully controlled to achieve desired profiles (Oxycyler™; Biospherix, Lacona, NY, USA). The CIH protocol adopted in the study has been described previously (McDonald et al., 2014, 2015b, 2016). Animals were placed in environmental chambers every morning in their conventional cages with free access to food and water for the duration of the treatment; they were housed in room air overnight. Chamber oxygen was cycled between normoxia (21%; 210 s) and hypoxia (5% at nadir; 90 s total) 12 times per hour. The intermittent bouts of hypoxia continued for 8 h per day for 21 consecutive days. Age-matched control (normoxia) groups were managed in parallel (*n* = 6 dams).

### Exposure to gestational CIH

Exposure to gCIH started within 24 h of identification of a copulation plug and continued until day 20 of gestation (*n* = 4 dams). Pregnant rats were housed in conventional cages together, until the last trimester when rats were separated for nesting; offspring were born in room air. The animals were then housed in the main rooms of our animal facility and weaned at postnatal day (P) 21, at which time males and females were divided and allocated for study on P22 or 42. All rats were housed in the main rooms of the animal facility until study day.

### Exposure to postnatal CIH

Pregnant rats allocated for pCIH exposure were housed in conventional cages together, until the last trimester when rats were separated for nesting; offspring were born in room air. Exposure to pCIH began within 24 h of birth [dams (*n* = 4) and respective litters were exposed]. The pups were weaned after the final day of treatment, with male and females divided and allocated for study on P22 or 42. The P42 groups were housed in the main rooms of our animal facility until experimental day. Data for sternohyoid muscle contractile function from these animals has been reported previously (McDonald et al., 2015b). We sought to examine the effects of exposure to gCIH or pCIH on diaphragm muscle function determined at the end of the postnatal period (P22) following standard weaning, or separately during young adulthood (P42). The time points are consistent with our previous studies (McDonald et al., 2015b, 2016) and were originally chosen based on their representation of important junctures in the developmental timeline and the provision of a normoxic recovery of 3 weeks' duration, equivalent to the CIH duration.

### Diaphragm muscle contractile function

Diaphragm muscle function was assessed *ex vivo* under isometric conditions using a standardized protocol as previously reported (McMorrow et al., 2011; Skelly et al., 2012; McDonald et al., 2014, 2015a,b, 2016; Shortt et al., 2014). Each experimental group at P22 (30–63 g) and P42 (112–290 g) consisted of rats randomized from multiple litters. Rats were killed by cervical dislocation following initial sedation with 5% isoflurane anesthesia. The diaphragm muscle was quickly excised and longitudinally arranged bundles prepared for assessment of contractile function. Muscle was suspended vertically with bone attached to a fixed tissue holder at one end and tendon attached using non-elastic string to a force transducer at the other end. Muscle baths were filled with Krebs salt solution and bubbled with control gas, 95% O_2_ and 5% CO_2_ initially and then either remained gassed with control gas or switched to bubbling with anoxic gas, 95% N_2_ and 5% CO_2_. The Krebs solution contained (mM): 120 NaCl, 25 NaHCO_3_, 12 MgSO_4_, 1.2 NaH_2_PO_4_, 2.5 calcium gluconate, 5 KCl, and 11.5 glucose; d-tubocurarine (25 μm) was used in all experiments to exclude any potential involvement of excitation of intramuscular nerve branches. Supramaximal square-wave constant current output (S48 Stimulator; Grass, Warwick, RI, USA) was used to stimulate the muscle via two platinum electrodes positioned at either side of the muscle in the bath. The change in tension was transduced, amplified and converted from an analog to digital signal, which was displayed and recorded using Chart software (AD Instruments, Oxford, UK) on a computer for later analysis. Optimal length was determined by eliciting multiple twitch force contractions following intermittent length changes until maximal force was achieved. Muscle twitch was elicited using single pulse (1 ms) stimulation. Muscle force was examined in response to increased frequency of stimulation (10, 20, 30, 40, 60, 80, 100 Hz), using 300 ms train duration and 2 min rest between each stimulus. Muscle fatigue was assessed by repeated 40 Hz stimulation once every 2 s for 5 min. Fatigue index was evaluated as measurement of force at 5 min expressed as % of initial force at time 0. In separate studies, diaphragm muscle function was assessed *ex vivo* in tissue baths gassed with 95% N_2_/5% CO_2_ to generate severe tissue hypoxia as previously described (McDonald et al., 2016). At the end of each experiment muscle was blotted dry and weighed. Tissue cross-sectional area was estimated using measurements of muscle length (cm), mass (g), and density (assumed to be 1.06 g.cm^−3^).

### Data analysis

Muscle twitch was examined for contraction time and half-relaxation time. Muscle force was normalized to cross-sectional area of the bundle and expressed as N/cm^2^. Fatigue was expressed at 5 min as a percentage of the initial force. Muscle force examined under severe hypoxic conditions *ex vivo* was expressed as percent of initial force under control conditions. Data were statistically analyzed using GraphPad Prism 6. Two-way ANOVA (gas × age) was performed with *post-hoc* Sidak tests. *P*-values are reported for all comparisons. Force-frequency curves were constructed allowing the determination of Hillslope and EF_50_ (stimulation frequency producing 50% of maximum force). Force-frequency curves were analyzed using repeated measures (RM)ANOVA. *P* < 0.05 was chosen as the criterion for statistical significance, however, the size of effect was primarily considered in the evaluation of the potential biological significance of the findings.

## Results

### Effects of CIH on body mass and hematocrit

Exposure to gCIH did not result in any difference in body mass or hematocrit of males or females at P22 or 42 compared with sham controls. Data for the effects of pCIH on body mass and hematocrit have been reported elsewhere (McDonald et al., 2015b).

### Single twitch force and contractile kinetics

Diaphragm contractile function was assessed at P22 and 42. Diaphragm single twitch force and contraction time were unaffected by age and antecedent exposure to CIH, both in male and female animals (Table 1). Half-relaxation time (HRT) was also unaltered by age and exposure to CIH in males. HRT was significantly altered by CIH in females (*P* = 0.04; Table 1), with prolongation of HRT in pCIH-exposed muscle, but *post-hoc* tests revealed no significant difference between sham and CIH groups.

**Table 1:** **Effects of early life exposure to CIH on diaphragm muscle twitch force and contractile kinetics in male and female rats under control conditions ***ex vivo*****.

	**Postnatal day 22**			**Postnatal day 42**			**Two-way ANOVA**
	**Sham (*n* = 10)**	**gCIH (*n* = 11)**	**pCIH (*n* = 8)**	**Sham (*n* = 9)**	**gCIH (*n* = 9)**	**pCIH (*n* = 7)**	
**MALE**							
Pt (N/cm^2^)	5.5 ± 1.8	4.1 ± 1.4	4.4 ± 1.4	4.7 ± 1.0	4.1 ± 1.4	5.1 ± 1.4	Gas *P* = 0.1; Age *P* > 0.9; Interaction *P* = 0.3
CT (ms)	30 ± 3	31 ± 4	31 ± 6	31 ± 8	28 ± 3	27 ± 4	Gas *P* = 0.7; Age *P* = 0.1; Interaction *P* = 0.3
HRT (ms)	31 ± 4	30 ± 6	27 ± 8	30 ± 5	27 ± 5	31 ± 6	Gas *P* = 0.7; Age *P* = 0.8; Interaction *P* = 0.3
**Female**							
Pt (N/cm^2^)	4.3 ± 1.4	4.5 ± 0.7	4.2 ± 1.1	4.7 ± 1.6	4.0 ± 0.9	4.0 ± 1.3	Gas *P* = 0.6; Age *P* = 0.8; Interaction *P* = 0.6
CT (ms)	29 ± 3	29 ± 4	34 ± 5	28 ± 6	27 ± 2	28 ± 3	Gas *P* = 0.2; Age *P* = 0.02; Interaction *P* = 0.4
HRT (ms)	29 ± 4	28 ± 5	35 ± 6	28 ± 5	28 ± 3	30 ± 4	Gas *P* = 0.04; Age *P* = 0.2; Interaction *P* = 0.5

*Data (mean ± SD) for twitch force (Pt), contraction time (CT), and half-relaxation time (HRT) of diaphragm muscle in male and female rats at postnatal day (P) 22 and P42 examined under control conditions ex vivo. Animals were exposed to sham (normoxia), gestational chronic intermittent hypoxia (gCIH), or postnatal CIH (pCIH). P-values following two-way ANOVA (gas × age) are reported*.

### Force-frequency relationship and peak tetanic force

**Figure 1:**
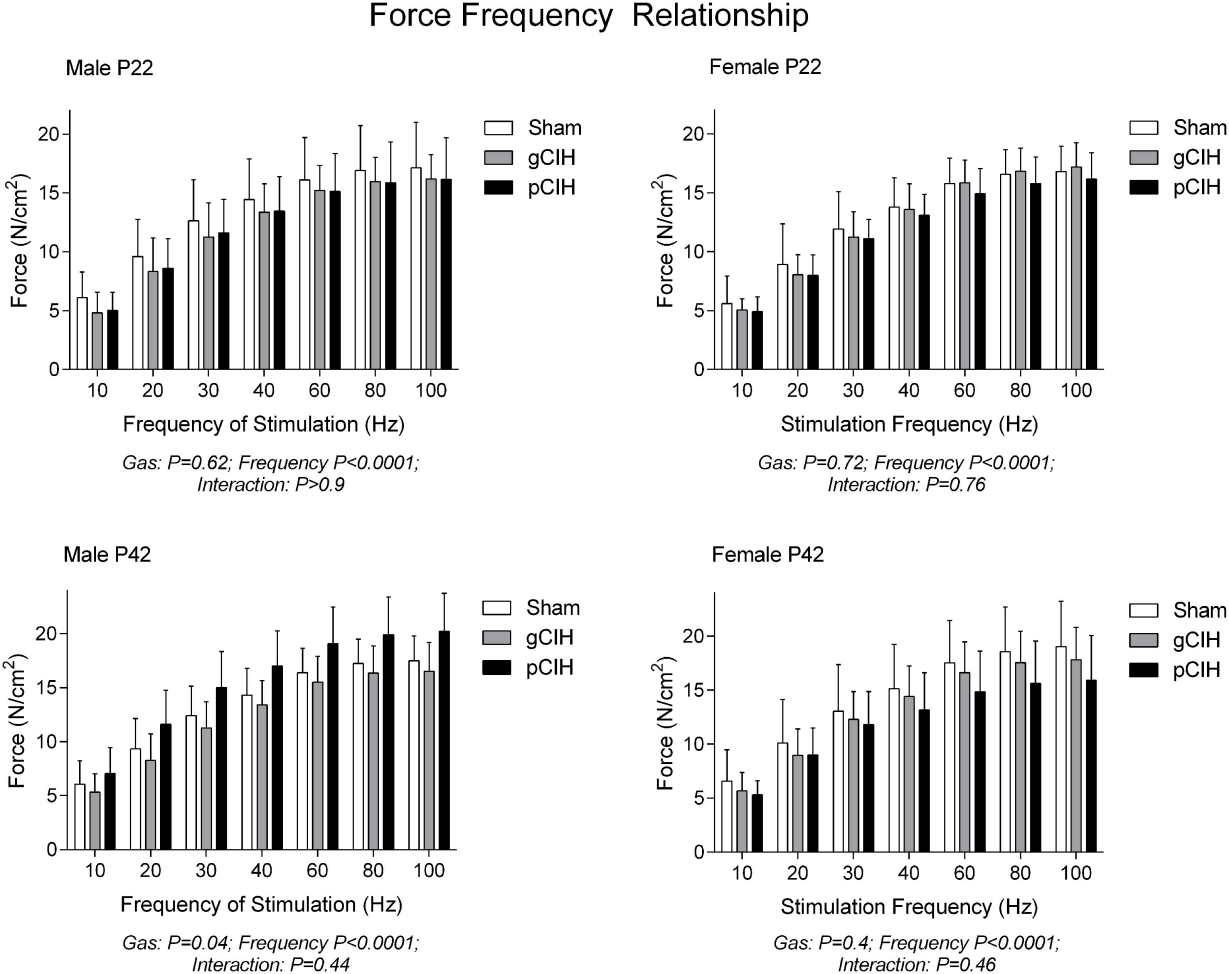
**Effects of early life exposure to CIH on diaphragm muscle force in male and female rats under control conditions ***ex vivo*****. Data (mean ± *SD*) for diaphragm muscle specific force across a range of stimulation frequencies in male (left panels) and female rats (right panels) at postnatal day (P) 22 and P42 examined under control conditions *ex vivo*. Animals were exposed to sham (normoxia), gestational chronic intermittent hypoxia (gCIH), or postnatal CIH (pCIH). *P*-values following two-way RMANOVA (gas × stimulation frequency) are reported. *Post-hoc* tests revealed no significant effect of gCIH or pCIH exposure on diaphragm force. *N*-values for animals as per Table 1.

Two-way RMANOVA (gas × stimulus frequency) revealed the expected increase in force with increased frequency of stimulation in all groups (Figure 1). At P22, in both males and females, there was no gas effect. At P42 in male but not female rats, two-way RMANOVA (gas × stimulus frequency) revealed a gas effect (*P* = 0.04), however *post-hoc* tests at each frequency did not reveal significant differences between sham and CIH-exposed groups. Moreover, key properties of the force-frequency relationship (Hillslope and EF_50_) were not altered by exposure to CIH in either sex (Table 2). Thus, early life exposure to CIH had no effect on diaphragm force-generating capacity.

**Table 2:** **Effects of early life exposure to CIH on diaphragm muscle force-frequency relationship in male and female rats under control conditions ***ex vivo*****.

	**Postnatal day 22**			**Postnatal day 42**			**Two-way ANOVA**
	**Sham (*n* = 10)**	**gCIH (*n* = 11)**	**pCIH (*n* = 8)**	**Sham (*n* = 9)**	**gCIH (*n* = 9)**	**pCIH (*n* = 7)**	
**MALE**							
Hillslope	2.4 ± 0.6	2.6 ± 1.0	2.2 ± 0.8	2.3 ± 0.6	2.7 ± 0.4	2.2 ± 0.4	Gas *P* = 0.1; Age *P* = 0.9; Interaction *P* = 0.9
EF_50_	24 ± 6	25 ± 6	22 ± 7	25 ± 7	27 ± 7	23 ± 4	Gas *P* = 0.1; Age *P* = 0.4; Interaction *P* > 0.9
**FEMALE**							
Hillslope	2.5 ± 0.6	2.6 ± 0.4	2.5 ± 0.4	2.1 ± 0.6	2.5 ± 0.4	2.2 ± 0.6	Gas *P* = 0.3; Age *P* = 0.1; Interaction *P* = 0.8
EF_50_	26 ± 9	29 ± 5	27 ± 3	26 ± 8	28 ± 4	25 ± 3	Gas *P* = 0.3; Age *P* = 0.8; Interaction *P* = 0.7

*Hillslope and EF_50_-values determined from force-frequency curves for diaphragm muscle in male and female rats at postnatal day (P) 22 and P42 examined under control conditions ex vivo. Animals were exposed to sham (normoxia), gestational chronic intermittent hypoxia (gCIH), or postnatal CIH (pCIH). R^2^-values for goodness of fit were >0.9. P-values following two-way ANOVA (gas × age) are reported*.

**Figure 2:**
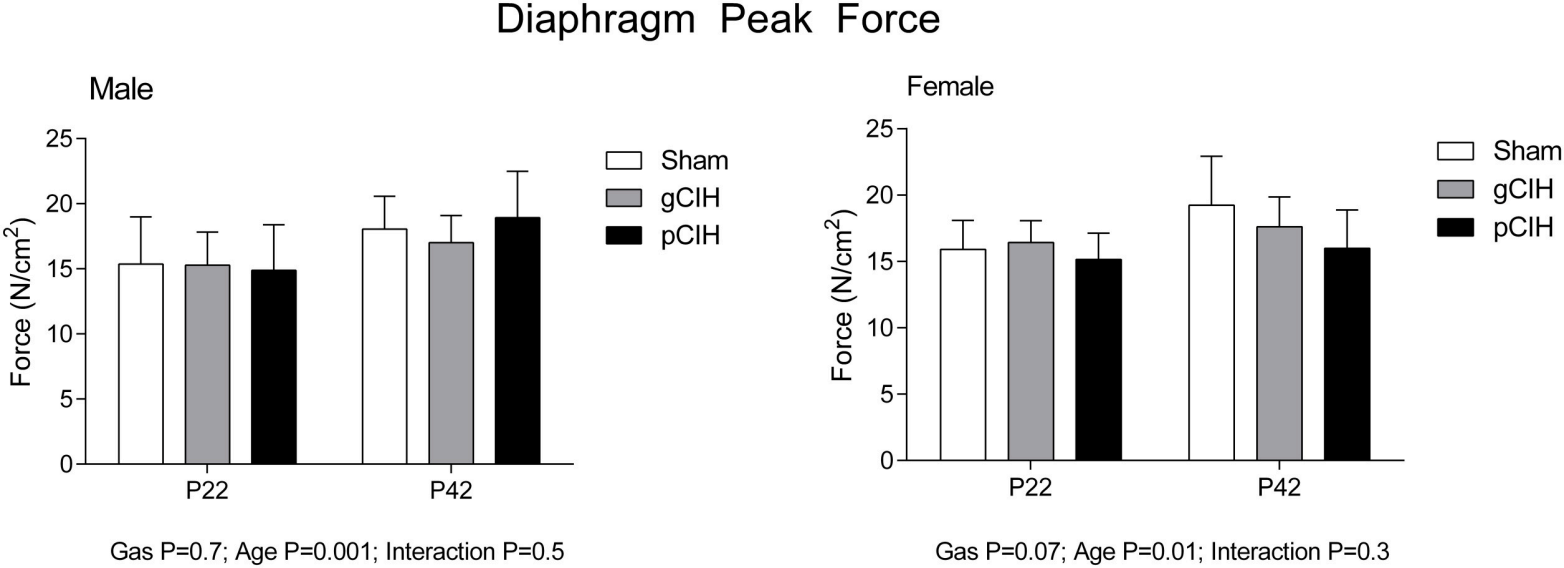
**Effects of early life exposure to CIH on diaphragm muscle force in male and female rats under hypoxic conditions ***ex vivo*****. Group data (mean ± *SD*) for peak diaphragm force in male and female rats at postnatal day (P) 22 and P42 examined under control conditions *ex vivo*. Animals were exposed to sham (normoxia), gestational chronic intermittent hypoxia (gCIH), or postnatal CIH (pCIH). *P*-values following two-way ANOVA (gas × age) are reported.

As expected, peak force at 100 Hz stimulation increased significantly with advancing age (Figure 2). Peak tetanic force was unaffected by antecedent exposure to CIH in male and female rats. To determine if a sex difference existed in the persistent effects of early life exposure to CIH on diaphragm force, two-way ANOVA (gas × sex) was performed for each age group. The analysis revealed that there was no sex difference at P22 or 42.

### Fatigue

**Figure 3:**
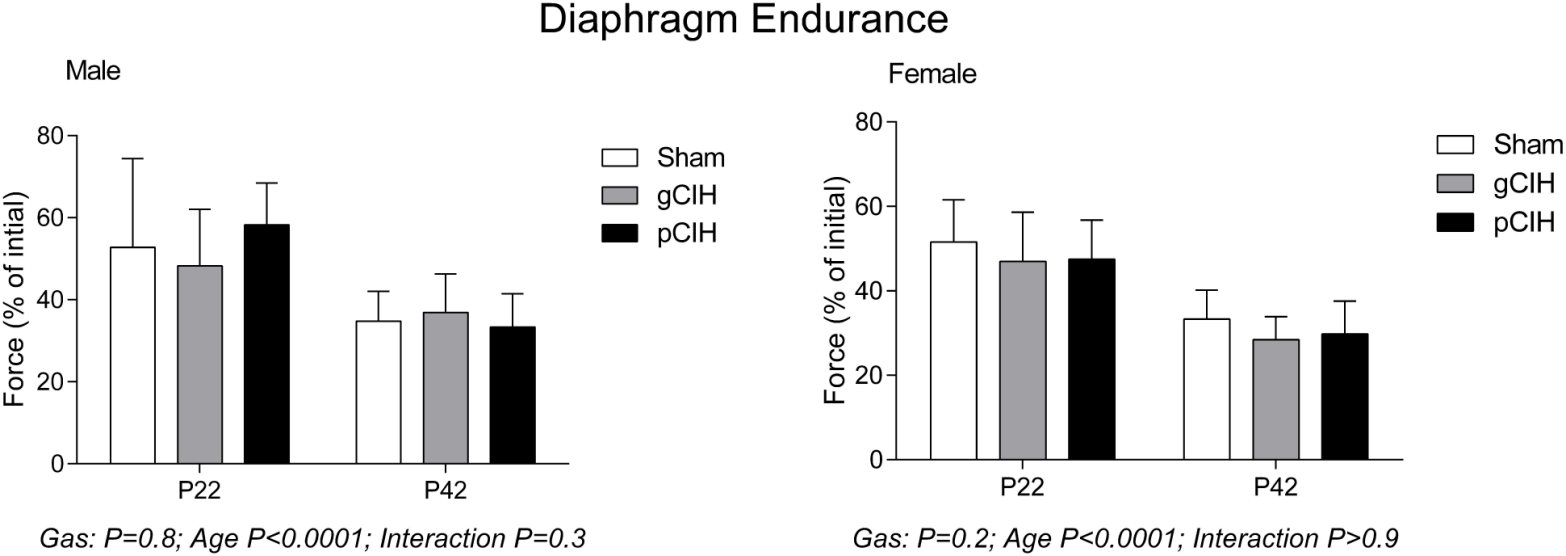
**Effects of early life exposure to CIH on diaphragm muscle fatigue in male and female rats under control conditions ***ex vivo*****. Fatigue index at 5 min (mean ± *SD*, % of initial force) of diaphragm muscle in male and female rats at postnatal day (P) 22 and P42 examined under control conditions *ex vivo*. Animals were exposed to sham (normoxia), gestational chronic intermittent hypoxia (gCIH), or postnatal CIH (pCIH). *P*-values following two-way ANOVA (gas × age) are reported. *N*-values for animals as per Table 1.

Fatigue index at 5 min (an index of endurance properties of diaphragm muscle in response to repeated stimulation, expressed as % of control force) decreased with age as expected i.e., diaphragm was more susceptible to fatigue with advancing age, but there was no effect of exposure to CIH on diaphragm muscle endurance in either sex (Figure 3).

### Hypoxic tolerance

Two-way ANOVA (gas × age) for single twitch force examined at P22, revealed a gas effect in male rats (*P* = 0.02), however *post-hoc* analysis did not reveal a significant difference between sham and CIH groups; moreover, when normalized to initial twitch force (under control conditions), there was no difference in the magnitude of hypoxic depression of force between sham and CIH groups for both male and female animals (Table 3).

**Table 3:** **Effects of early life exposure to CIH on diaphragm muscle twitch force and contractile kinetics in male and female rats under hypoxic conditions ***ex vivo*****.

	**Postnatal22**			**Postnatal42**			**Two-way ANOVA**
	**Sham (*n* = 7)**	**gCIH (*n* = 7)**	**pCIH (*n* = 6)**	**Sham (*n* = 9)**	**gCIH (*n* = 8)**	**pCIH (*n* = 8)**	
**MALE HYPOXIA**							
Pt (N/cm^2^)	2.9 ± 1.1	3.2 ± 0.7	2.2 ± 0.8	2.3 ± 0.9	3.1 ± 0.8	2.3 ± 0.7	Gas *P* = 0.02; Age *P* = 0.4; Interaction *P* = 0.5
Pt (% of initial)	82 ± 12	76 ± 6	73 ± 11	71 ± 19	72 ± 12	78 ± 13	Gas *P* = 0.7; Age *P* = 0.2; Interaction *P* = 0.2
CT (ms)	24 ± 3	23 ± 2	25 ± 4	21 ± 4	22 ± 3	19 ± 2	Gas *P* = 0.98; Age *P* = 0.004; Interaction *P* = 0.1
HRT (ms)	23 ± 4	23 ± 2	20 ± 4	21 ± 5	21 ± 3	20 ± 4	Gas *P* = 0.4; Age *P* = 0.2; Interaction *P* = 0.8
**FEMALE HYPOXIA**							
Pt (N/cm^2^)	2.7 ± 0.6	2.9 ± 0.5	3.1 ± 0.4	2.5 ± 0.8	2.7 ± 0.9	2.5 ± 0.9	Gas *P* = 0.6; Age *P* = 0.1; Interaction *P* = 0.6
Pt (% of initial)	77 ± 12	78 ± 7	79 ± 7	71 ± 16	72 ± 20	82 ± 10	Gas *P* = 0.5; Age *P* = 0.6; Interaction *P* = 0.7
CT (ms)	26 ± 6	25 ± 4	24 ± 2	19 ± 3	20 ± 3	20 ± 5	Gas *P* = 0.9; Age *P* = 0.0005; Interaction *P* = 0.7
HRT (ms)	25 ± 4	26 ± 4	25 ± 6	22 ± 5	25 ± 14	21 ± 6	Gas *P* = 0.7; Age *P* = 0.3; Interaction *P* = 0.9

*Experimental data (mean ± SD) for absolute twitch force (Pt) (and Pt expressed as percentage of initial twitch in control conditions), contraction time (CT), and half-relaxation time (HRT) of diaphragm muscle in male and female rats at postnatal day (P) 22 and P42 examined under hypoxic conditions ex vivo. Animals were exposed to sham (normoxia), gestational chronic intermittent hypoxia (gCIH), or postnatal CIH (pCIH). P-values following two-way ANOVA (gas × age) are reported*.

**Figure 4:**
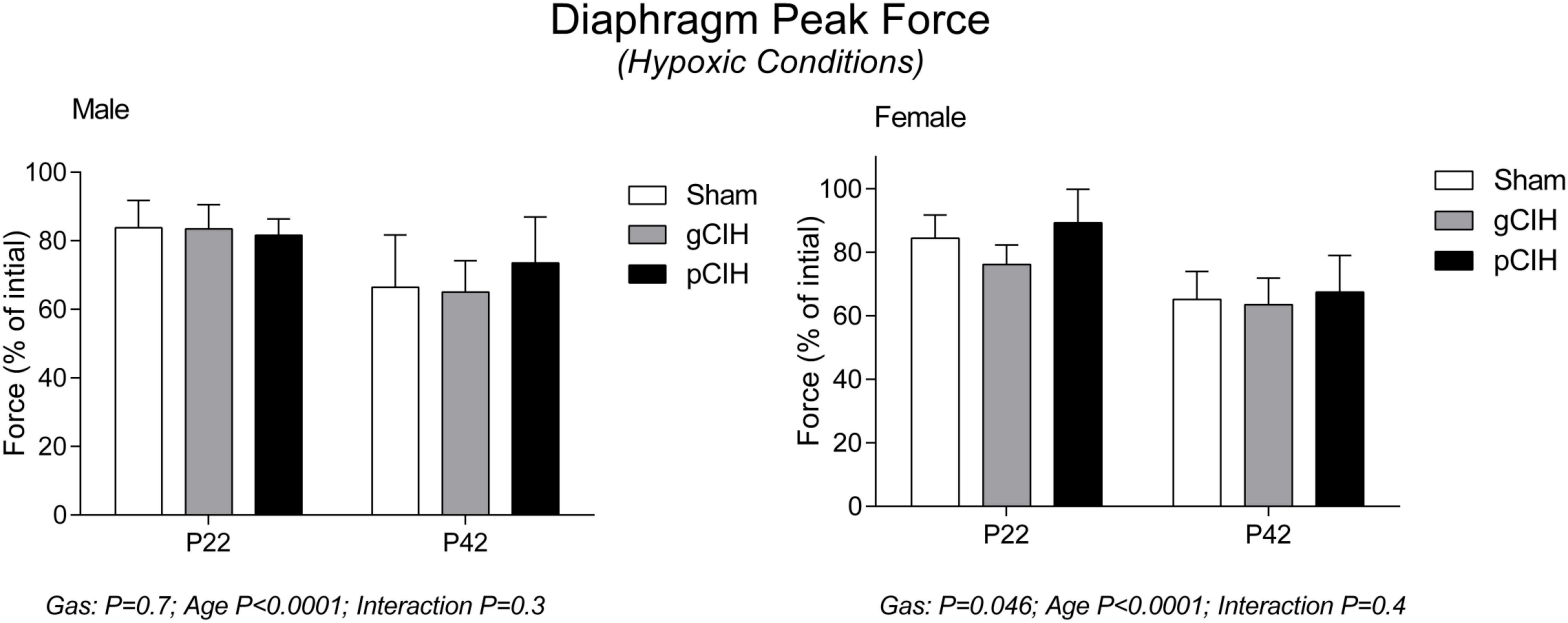
**Effects of early life exposure to CIH on diaphragm muscle force in male and female rats under hypoxic conditions ***ex vivo*****. Group data (mean ± *SD*) for peak diaphragm force expressed as percentage of initial peak diaphragm force (in control conditions) in male and female rats at postnatal day (P) 22 and P42 examined under hypoxic conditions *ex vivo*. Animals were exposed to sham (normoxia), gestational chronic intermittent hypoxia (gCIH), or postnatal CIH (pCIH). *P*-values following two-way ANOVA (gas × age) are reported.

Early life exposure to CIH did not affect diaphragm force-generating capacity in male rat diaphragm muscle studied under hypoxic conditions *ex vivo* (Figure 4). In females, two-way ANOVA (gas × age) revealed a significant effect of exposure to CIH, but *post-hoc* tests did not reveal a significant difference between sham and CIH groups (Figure 4). Force-frequency relationship of diaphragm muscle under hypoxic conditions was unaffected by exposure to CIH, although EF_50_ under hypoxic conditions was right-shifted in both male and female rats with advancing age (*P* < 0.05; Table 4).

**Table 4:** **Effects of early life exposure to CIH on diaphragm muscle force-frequency relationship in male and female rats under hypoxic conditions ***ex vivo*****.

	**Postnatal day 22**			**Postnatal day 42**			**Two-way ANOVA**
	**Sham (*n* = 8)**	**gCIH (*n* = 8)**	**pCIH (*n* = 7)**	**Sham (*n* = 9)**	**gCIH (*n* = 8)**	**pCIH (*n* = 8)**	
**MALE HYPOXIA**							
Hillslope	3.2 ± 0.9	2.9 ± 0.6	2.7 ± 0.5	3.4 ± 1.7	2.9 ± 0.5	2.6 ± 0.4	Gas *P* = 0.2; Age *P* = 0.9; Interaction *P* = 0.9
EF_50_	35 ± 5	39 ± 7	34 ± 4	44 ± 12	40 ± 7	44 ± 4	Gas *P* = 0.9; Age *P* = 0.004; Interaction *P* = 0.2
**FEMALE HYPOXIA**							
Hillslope	3.1 ± 0.7	2.8 ± 0.4	2.4 ± 0.5	2.9 ± 0.7	2.6 ± 0.9	2.6 ± 0.6	Gas *P* = 0.1; Age *P* = 0.7; Interaction *P* = 0.6
EF_50_	35 ± 6	37 ± 7	31 ± 5	42 ± 8	46 ± 9	42 ± 10	Gas *P* = 0.2; Age *P* = 0.0004; Interaction *P* = 0.8

*Hillslope and EF_50_-values determined from force-frequency curves for diaphragm muscle in hypoxia in male and female rats at postnatal day (P) 22 and P42 examined under hypoxic conditions ex vivo. Animals were exposed to sham (normoxia), gestational chronic intermittent hypoxia (gCIH), or postnatal CIH (pCIH). R^2^-values for goodness of fit were >0.9. P-values following two-way ANOVA (gas × age) are reported*.

**Figure 5:**
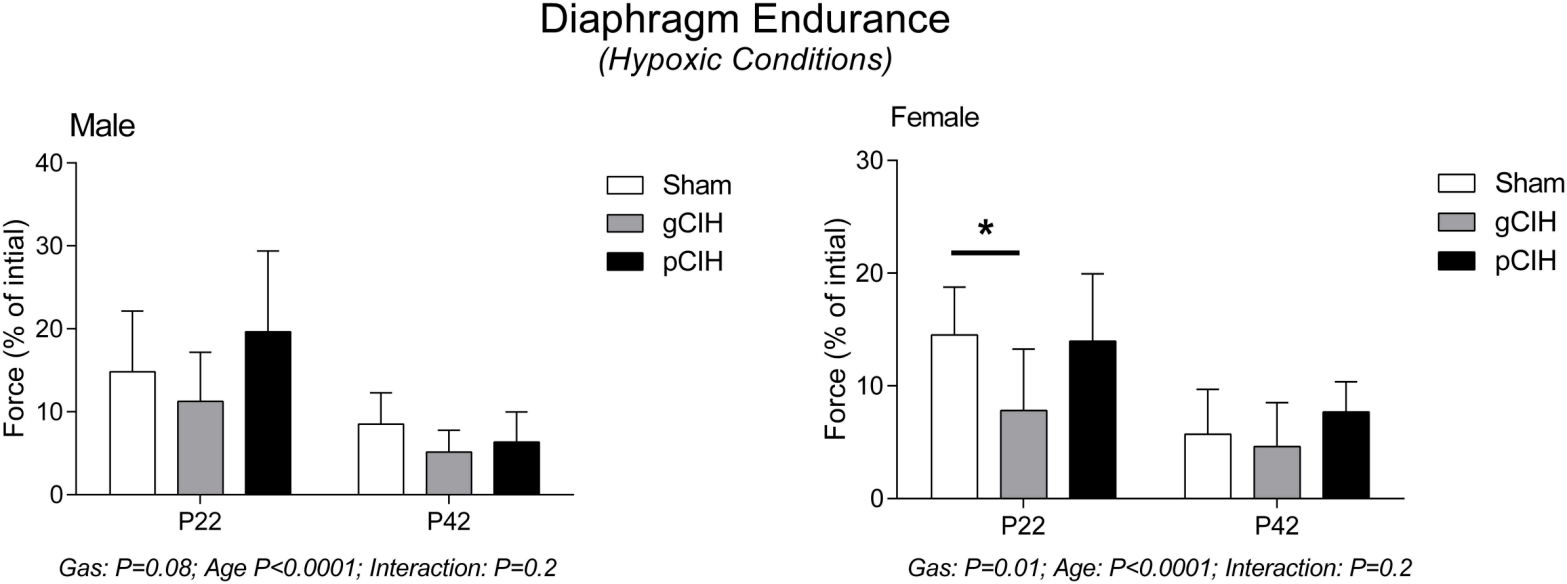
**Effects of early life exposure to CIH on diaphragm muscle fatigue in male and female rats under hypoxic conditions ***ex vivo*****. Fatigue index at 5 min (mean ± *SD*) of diaphragm muscle in male and female rats at postnatal day (P) 22 and P42 examined under hypoxic conditions *ex vivo*. Animals were exposed to sham (normoxia), gestational chronic intermittent hypoxia (gCIH), or postnatal CIH (pCIH). *P*-values following two-way ANOVA (gas × age) are reported. ^*^P < 0.05 Sidak *post-hoc* test.

Diaphragm endurance under hypoxic conditions was considerably less compared with control conditions and decreased with age in both male and female rats (Figure 5). Fatigue index during hypoxia *ex vivo* was decreased slightly following exposure to gCIH compared with sham, and this was significant in females at P22 (*P* = 0.01).

## Discussion

The principal finding of this study is that early life exposure to CIH has no effect on diaphragm muscle force-generating capacity. Neither gestational CIH nor postnatal CIH affected diaphragm force or endurance properties in either male or female animals. Similarly, hypoxic tolerance in diaphragm muscle of both sexes was unaffected by antecedent hypoxic exposure. The study extends and complements a recent report by our group, which observed that diaphragm function in adulthood is unaffected by postnatal exposure to CIH (McDonald et al., 2016). The findings in diaphragm muscle contrast with our observations in sternohyoid muscle from the same animals (McDonald et al., 2015b). Thus, differential effects of early life exposure to hypoxia are expressed in sternohyoid and diaphragm muscle, with evidence of weakness in the airway dilator muscle that persists for several weeks upon return to normoxia (McDonald et al., 2015b) and increased susceptibility to subsequent hypoxic insult (McDonald et al., 2016), whereas no functional impairment is evident in diaphragm muscle at any stage, revealing an apparent relative resilience in rat diaphragm muscle to hypoxic stress in early life (McDonald et al., 2015b, 2016; this study). The latter is consistent with observations of sternohyoid, but not diaphragm, dysfunction following chronic sustained hypoxia during development in rats (Carberry et al., 2014), and some reports in adult rats documenting no functional impairment in diaphragm following chronic sustained hypoxia (El-Khoury et al., 2003; McMorrow et al., 2011; Gamboa and Andrade, 2012).

Our study is the first report of the effect of early life exposure to CIH on diaphragm function. Though some subtle effects of CIH exposure on diaphragm muscle performance were evident, on balance we conclude that early life exposure to CIH has no major effect on diaphragm force in either sex, at least under the conditions established in our study. We do not discount the possibility that CIH has adverse consequences for diaphragm muscle performance *per-se*. Indeed, diaphragm weakness and fatigue was reported in adult male rats exposed to CIH, an outcome dependent on the duration and intensity of the hypoxic stimulus (Shortt et al., 2014). Moreover, chronic sustained hypoxia is reported to cause diaphragm weakness, an effect dependent on the timing of presentation of the stimulus during the developmental period (Kass and Bazzy, 2001), consistent with observations of sternohyoid muscle dysfunction following chronic sustained hypoxia (Carberry et al., 2014). Whilst, the entire gestational and neonatal period is generally regarded as a relatively vulnerable period wherein the respiratory control system is quite malleable, we acknowledge that within the timeframe of our study there may well have been periods of maladaptive remodeling leading to dysfunction that in turn were countered by adaptive or compensatory responses. There are likely complex temporal responses to hypoxia in developing respiratory muscle and our study design was focused on just two distinct end-points (P22 and 42). In particular, the first week of life in the rat is known to be especially sensitive to perturbation with consequences for cardiorespiratory control (Bavis, 2005; Bavis et al., 2007; Pozo et al., 2012; Fournier et al., 2015). It would be interesting to explore the effects of CIH on respiratory muscle performance during this period i.e., functional assessment immediately following exposure to CIH during the first week of life. The latter may have greater relevance to preterm infants who experience CIH resulting from apnea of prematurity. Similarly, earlier assessment of diaphragm muscle performance following exposure to gCIH (e.g., during the first week of life) might have revealed evidence of maladaptive plasticity with functional consequences. Our initial study point at P22 was 3 weeks after termination of the gCIH exposure, potentially providing a window for recovery during the rapidly changing developmental timeline (O'Connell et al., 2013). Thus, further work is required to comprehensively determine the consequences of exposure to early life CIH on diaphragm muscle form and function, but the evidence to hand at this juncture suggests no major lasting effect of early life CIH on diaphragm performance. Of note in the present study, exposure to gCIH increased diaphragm fatigue during severe hypoxia at P22 in female but not male animals. The effect is small but might point to increased susceptibility to respiratory impairment under such conditions should they present *in vivo*. The observation suggests that further evaluation of hypoxic tolerance and potential differences between the sexes following exposure to gCIH are warranted.

It is important to consider the findings of this study in the context of our previous observations revealing sternohyoid muscle weakness in pCIH-exposed rats with lasting effects leading to increased susceptibility to hypoxic insult in adulthood. The co-ordinated activation of pharyngeal upper airway muscles (e.g., sternohyoid) and thoracic muscles (e.g., diaphragm) optimizes ventilation and is critical for airway stability. The sternohyoid appears more vulnerable to hypoxia-induced force decline (McDonald et al., 2015b, 2016), perhaps due to its composition of fast muscle fiber types compared to predominantly slow fiber types of the diaphragm (O'Connell et al., 2013). Differential functional responses to hypoxia in fast and slow limb muscles has been observed (Howlett and Hogan, 2007). A study of the structure and function of rat sternohyoid and diaphragm muscle during the first month of life revealed a faster maturation rate in the sternohyoid compared with the diaphragm (O'Connell et al., 2013). Fiber remodeling may partly explain pCIH-induced sternohyoid weakness (McDonald et al., 2015b, 2016), and as such intrinsic differences in fiber complement between sternohyoid and diaphragm, with related differences in metabolic strategies (glycolytic vs. oxidative), combined with the different functional requirements of the two muscles might provide a substrate for maladaptive remodeling in one muscle (sternohyoid), but not the other (diaphragm). It is also plausible to consider that the diaphragm may be intrinsically protected and more robust in response to adversity because of its essential role in respiratory homeostasis. Redox remodeling appears pivotal to CIH-induced respiratory muscle dysfunction (Williams et al., 2015), and antioxidant supplementation has been shown to ameliorate or prevent CIH-induced weakness and fatigue (Dunleavy et al., 2008; Skelly et al., 2012; Shortt et al., 2014). Differential redox responses in diaphragm and sternohyoid could plausibly underpin the apparent difference in muscle responses to CIH-related stress.

Whatever the mechanism, a mismatch in the performance of diaphragm and upper airway dilator muscles potentially places the compliant pharyngeal airway at risk. As such, weakened airway dilator muscle activity in the face of normal pump muscle function threatens airway caliber, at least in species such as human with collapsible upper airways. Thus, our findings may have relevance to the control of airway patency in humans, where maintenance of diaphragm muscle force with coincident depressed upper airway muscle force would increase the risk of airway collapse. Weak upper airway muscles may not adequately counteract the negative pressure generated by the diaphragm during inspiration, and/or may not be able to recover a collapsed pharyngeal airway with persistent diaphragm contraction.

In summary, the present study demonstrates that exposure to CIH during early life has little effect on diaphragm force or fatigue. Differential effects of early life CIH on airway dilator and diaphragm muscle performance may result in a mismatch with deleterious consequences for the control of airway caliber. As such, extrapolating (with caution) the findings to humans, we speculate that early life hypoxic stress may have long-lasting implications for the control of airway patency and respiratory homeostasis. In this way the findings suggest that early life adversity characterized by CIH may have implications for the development or worsening of obstructive sleep apnea in later life.

## Author contributions

FM and KO designed the study. FM conducted experiments and analyzed data. FM and KO interpreted data sets and performed statistical analyses. All authors contributed to the writing of the manuscript.

## Funding

Funded by Health Research Board (Ireland). FM was funded by the School of Medicine and Medical Science, University College Dublin, Ireland.

### Conflict of interest statement

The authors declare that the research was conducted in the absence of any commercial or financial relationships that could be construed as a potential conflict of interest.
